# The *Non-Coding RNA* Journal Club: Highlights on Recent Papers—13

**DOI:** 10.3390/ncrna9060076

**Published:** 2023-12-14

**Authors:** Patrick K. T. Shiu, Johanna K. DiStefano, Suresh K. Alahari, Francisco J. Enguita, Mark W. Feinberg, Nikolaos Sideris, Salih Bayraktar, Leandro Castellano, Diana Luna Buitrago, Andrea Caporali, Alessandro Mannucci, Ajay Goel

**Affiliations:** 1 Division of Biological Sciences, University of Missouri, Columbia, MO 65211, USA; 2 Metabolic Disease Research Unit, Translational Genomics Research Institute, Phoenix, AZ 85004, USA; 3 Department of Biochemistry and Molecular Biology, LSUHSC School of Medicine, New Orleans, LA 70112, USA; 4 Instituto de Medicina Molecular João Lobo Antunes, Faculdade de Medicina, Universidade de Lisboa, 1649-028 Lisboa, Portugal; 5 Division of Cardiovascular Medicine, Brigham and Women’s Hospital, Harvard Medical School, Boston, MA 02115, USA; 6 School of Life Sciences, University of Sussex, Falmer, Brighton BN1 9QG, UKsb549@sussex.ac.uk (S.B.); 7 Department of Surgery and Cancer, Imperial Centre for Translational and Experimental Medicine (ICTEM), Imperial College London, London W12 0NN, UK; 8 BHF Centre for Cardiovascular Science, The Queen’s Medical Research Institute, University of Edinburgh, Edinburgh EH16 4TJ, UK; d.luna-buitrago@sms.ed.ac.uk; 9 Gastroenterology and Gastrointestinal Endoscopy Unit, IRCCS San Raffaele Scientific Institute, Vita-Salute San Raffaele University, 20132 Milan, Italy; amannucci@coh.org; 10 Department of Molecular Diagnostics and Experimental Therapeutics, Beckman Research Institute of City of Hope, Biomedical Research Center, Monrovia, CA 91016, USA; 11 City of Hope Comprehensive Cancer Center, Duarte, CA 91010, USA

## 1. Introduction

We are delighted to share with you our thirteenth Journal Club and highlight some of the most interesting papers published recently. We hope to keep you up to date with non-coding RNA research that extends beyond your study area. The *Non-Coding RNA* Scientific Board wishes you an exciting and fruitful read.

## 2. *Trans*-Species microRNA Genes from a Parasitic Plant Share a Promoter Element

### Highlight by Patrick K. T. Shiu

*Trans*-species small RNAs are those that move from one organism to another. While these exported RNAs have been gaining attention in the past few years, how they are regulated is still very much unexplored. In a recent article of *The Plant Cell*, Hudzik and others showed that *trans*-species microRNAs from a parasitic plant are regulated differently from other canonical microRNAs [1].

The parasitic plant *Cuscuta campestris* (dodder) is photosynthetically inactive, and it feeds on other plants with penetrating structures called haustoria. At the host–parasite interface, *C. campestris* accumulates a set of interface-induced microRNAs (IIMs) that could modulate host gene expression for its advantage. In this work, the authors showed that IIMs start appearing in early haustorium development (before host penetration) and that their induction does not vary between different host species. Furthermore, IIMs can be found in haustoria artificially stimulated without an adjacent host. These observations suggest that the production of IIMs is guided by a host-independent, intrinsic program and that they are primed for immediate deployment in a developing haustorium.

While canonical plant microRNAs originate from RNA Polymerase II (Pol II) and do not have any common promoter elements other than a TATA box, the majority (93%) of IIM loci contain a 10-nucleotide upstream sequence element (USE) that is also found in a group of small nuclear RNA (U-snRNA) genes. Depending on how far this USE is upstream of the transcriptional start site (TSS), it can drive either Pol II (66–67 bp upstream) or Pol III (54–55 bp upstream) transcription of U-snRNAs. The USE-TSS distance peaks at ~55 bp for IIM loci, suggesting that they are transcribed by Pol III. Features of the IIM primary transcripts (5′ triphosphate ends with no caps and nonpolyadenylated 3′ ends terminated at polyT sites) agree with their having a Pol III origin. According to a heterologous experiment, the USE appears to be an essential *cis*-acting factor for IIM expression.

This is the first time Pol III has been shown to engage in the production of plant microRNAs. It is possible that the involvement of Pol III could somehow tag IIMs for their specialized processing and export to the host. Future studies should shed light on whether other parasitic plants also utilize this *trans*-species microRNA strategy and how we can exploit it for pest control.

## 3. NEAT1_2 RNA Domains Define the Independence of Paraspeckles within the Nucleus

### Highlight by Johanna K. DiStefano

Membraneless organelles (MLOs) form through phase separation, concentrating biomolecules in distinct compartments. While these MLOs coexist within cells, the mechanisms by which they preserve their autonomy, avoiding coalescence or engulfment, are not well understood. Specifically, it is unclear how paraspeckles (PSs) and nuclear speckles (NSs) maintain independence despite their close proximity. In a recent issue of *Nature Cell Biology*, Takakuwa et al. [2] demonstrated that specific shell-localizing proteins, determined by RNA domains within the long non-coding RNA NEAT1_2, play a key role in ensuring the autonomy of PSs from NSs.

In this study, mutant NEAT1_2-derived paraspeckles, termed mini-PSs, were incorporated into NSs, challenging the conventional understanding of MLO independence. Despite possessing a core–shell organization, mini-PSs lost their ability to exist as distinct MLOs and were unexpectedly occluded by NSs. Specific NEAT1_2 RNA domains, particularly in the 1–8 kb and 16.6–20.2 kb regions, were essential for orchestrating the segregation process. Additionally, the reduced recruitment of proteins such as SFPQ, TDP-43, HNRNPH1, HNRNPF, and BRG1 suggested their involvement in the segregation mechanism. The co-transcriptional formation of NEAT1_2 ribonucleoproteins antagonized the association of splicing factors with NEAT1 lncRNAs, contributing to the construction of PSs with a proper core–shell architecture. De novo-formed mini-PSs were incorporated into NSs while retaining their internal core–shell structure. Artificial tethering of U2 snRNP-related proteins induced significant internalization of PSs into NSs, underscoring the significance of shell localization and emphasizing the role of SFPQ, HNRNPF, and BRG1 in controlling NS or nucleoplasmic localization.

This work provides crucial insights into MLO dynamics and the regulatory role of lncRNAs within these organelles. The study underscores the pivotal role of specific NEAT1_2 RNA domains and paraspeckle shell components in dictating the independence of MLOs within the nucleus. The unexpected incorporation of mini-PSs into nuclear speckles sheds light on the intricate dynamics of MLO interactions, paving the way for future research on the regulatory networks governing different MLO interactions.

## 4. Circulating microRNAs Help in Identification of BRCA1/2 Mutation Carriers

### Highlight by Suresh K. Alahari

BRCA1 and BRCA2 genes play an important role in hereditary breast and ovarian cancers. These genes regulate DNA repair via homologous recombination. Failure of homologous recombination causes DNA repair problems, which lead to mutagenesis. Thus, detection of BRCA1/BRCA2 mutation carriers assist in the prevention or stopping of breast and ovarian cancers. It has been shown that microRNAs regulate BRCA-mediated DNA repair [3]. Identification of specific circulating microRNAs in BRCA1/2 mutated patients will help detect the disease early, and this is a non-invasive approach which can be tested using a small blood sample.

In the current study, they tested microRNA expression in sera from 653 subjects that include 350 with BRCA1 and BRCA2 mutations and 303 with wild type [3]. The samples were collected from five international locations, including Tata Medical Center, India, Dana-Farber Cancer Institute, Boston, USA, University of Pennsylvania, Philadelphia, USAP, and Pomeranian Medical University, Poland. Here, the authors used RNA sequencing for miRNA quantification and developed models that differentiate subjects with BRCA1/2 mutations from BRCA wild type. The analysis revealed that 19 microRNAs were associated with BRCA1/2 mutations. Further logistic regression model analysis narrowed the number of microRNAs to 10; these include miR-20b-5p, miR-19b-3p, let-7b-5p, miR-320b, miR-139-3p, miR-30D-5p, miR-17-5P, miR-182-5p, miR-421, and miR-375-5p. Thus, these microRNAs will serve as predictable markers for BRCA1/2 mutations positivity, and these will serve as liquid biopsy markers.

The authors admitted this study has some weaknesses as well. These include the following: (1) the data are generated based on next generation sequencing (NGS), which is an expensive approach; (2) the usage of different sequencing platforms; (3) the associations were not tested for other DNA repair defects; and (4) correlation was not conducted with different racial and ethnic populations.

## 5. A New Function for an Old Friend: DICER Nuclease and R-Loop Resolution

### Highlight by Francisco J. Enguita

R-loops are three-stranded nucleic acid structures formed by the interaction of DNA, RNA, and the displaced single-stranded DNA. While R-loops play roles in normal cellular processes, their persistence or improper regulation can lead to genome instability. The displaced single-stranded DNA in R-loops is more susceptible to damage, and unresolved R-loops can contribute to genomic rearrangements and DNA breaks. To counteract the accumulation of R-loops, cells have different mechanisms, including the prevention RNA-DNA hybridization by RNA biogenesis factors, the involvement of helicases to unwind the RNA–DNA hybrids, or the degradation of the hybrid by specific nucleases like RNase H. A recent manuscript by Camino and coworkers described how a key enzyme involved in miRNA biogenesis, DICER, is able to recognize the RNA–DNA hybrids acting as a resolving factor that specifically cleaves the RNA molecule within the hybrid [4]. DICER counteracts the accumulation of nuclear R-loops independently of its cytoplasmic activity on miRNA biogenesis and the RNA interference (RNAi) machinery. According to Camino and coworkers’ data, DICER binds R-loops with high affinity in vitro and cleaves the RNA component of DNA–RNA hybrids in R-loops. These results indicate that the RNase activity of DICER promotes genome stability in eukaryotes and characterizes this nuclease as a multi-functional factor on the interface of genome stability and the regulation of gene expression [4].

## 6. Heterogeneity of Alternatively Expressed lncRNA Impacts Penetrance for the Strongest-Linked SNP in Coronary Artery Disease

### Highlight by Mark W. Feinberg

Coronary artery disease (CAD) remains a major contributor to cardiovascular mortality worldwide. Accumulating genome-wide association studies (GWAS) demonstrate that unique single-nucleotide polymorphisms (SNPs) are linked to increased risk of CAD. A multitude of SNPs are found in the non-coding genome including regions associated with lncRNAs. SNPs with the strongest association with CAD are found in the non-coding 9p21.3 locus, which includes the lncRNA *ANRIL* [5]. This association is unaltered even after adjustment for traditional cardiovascular risk factors [6]. Interestingly, patients harboring this SNP may exhibit variable vascular disease pathology. Like many lncRNAs, ANRIL exists with multiple isoforms and can be expressed in different cell types, raising the question of how do SNPs affecting ANRIL isoforms link to the complex biology of CAD?

In the journal *PNAS*, Mayner et al. [7] use human-induced pluripotent stem-cell-derived vascular smooth muscle cells (iPSC-VSMCs) to identify significant heterogeneity of isoforms and variable ANRIL penetrance that may underlie the mixed functional outcomes that interplay with the complex CAD pathology. The categorization of risk haplotypes demonstrated marked differences in iPSC-VSMCs morphology and functional properties, including proliferation, contraction, and adhesion. Short isoforms of *ANRIL* were expressed in weakly adherent iPSC-VSMCs compared to stronger adherent cells. Subsequent studies demonstrated that this phenotype is due to variability of *ANRIL* isoforms containing a proximal transcription termination in exon 13. Upon overexpression of this isoform in knockout cells, ANRIL inhibited VSMC adhesion, contractility, and VSMC marker expression, effects that would be anticipated to confer a more proliferative, migratory synthetic phenotype typically observed in atherosclerosis. Indeed, there is an overabundance of VSMCs in atherosclerotic lesions of patients that harbor the 9p21 risk haplotype, albeit with some variability [8]. Collectively, this elegant study revealed that heterogeneity in alternatively expressed lncRNA isoforms may underlie the variability of penetrance of the disease phenotype for at-risk patients. Future studies will be of interest with regard to how high-risk disease-linked SNPs mechanistically give rise to lncRNA alternative splicing and differential expression in cell-specific populations.

## 7. LncRNA Sweetheart, an RNA to Mend a Broken Heart

### Highlight by Nikolaos Sideris, Salih Bayraktar, and Leandro Castellano

In the article by Sandra Rogala et al. [9] in *Nature Communications*, the researchers describe the role of lncRNA Sweetheart (*Swhtr*) in modulating compensatory cardiac hypertrophy following myocardial injury in murine males. The *Swhtr* sequence is in the same region as that of the transcription-factor-coding gene Nkx2-5 and is expressed by it specifically in the heart. While *Swhtr* appears to be expressed at lower levels than Nkx2-5, they both exhibit similar patterns of expression at different stages of heart development. Fractionation of cardiomyocytes followed by qPCR and smFISH showed the lncRNA to be chromatin bound, localizing at the site of its transcription. The authors then generated a *Swhtr*-KO mouse line to investigate its function and found that loss of *Swhtr* does not affect Nkx2-5 expression or heart development/function under normal conditions. This observation was further confirmed via ASO-mediated knockdown. Interestingly, upon induction of myocardial infarction, a higher mortality rate was observed in the *Swhtr* null mice, despite similar-sized infarctions and no differences in measured heart parameters. Strikingly, the authors found the null mice to lack compensatory thickening in the interventricular septum compared to the wild-type mice. Further experiments were conducted in a *Swhtr* rescue line, which showed similarities to the wild-type mice in all terms. This is a testament to the significance of *Swhtr* to the early stress and adaptive response of cardiac tissue upon myocardial infarction. Upon studying the expression profiles of primary cultures from heart tissue slices under normal conditions and conditions mimicking the infarction model, they found minimal changes in wild-type cells, while multiple genes were dysregulated in the *Swhtr* null cells. While Nkx2-5 expression is unaffected by loss of *Swhtr*, almost all dysregulated genes showed Nkx2-5 occupancy in ChIP-seq data. This, combined with *Swhtr* and Nkx2-5 co-precipitation in pulldown experiments, along with results from smFISH, suggest that this lncRNA and transcription factor interact to regulate the cardiac stress response.

## 8. Characterization of Non-Coding RNA Expression within Aged and Rejuvenated Mice to Reveal Potential Targets for New Pharmaceutical Approaches

### Highlight by Diana Luna Buitrago and Andrea Caporali

Aging and the associated underlying processes are considered a significant risk factor for a range of diseases, including dementia, diabetes, cancer, and atherosclerosis. The complex mechanisms defining aging are poorly understood despite the clear biological significance. It has been acknowledged over the years that non-coding RNAs are considered interesting targets due to their involvement in age-related processes such as intercellular communication and epigenetics.

In a recent paper in *Nature Biotechnology*, Keller et al. addressed the lack of available transcriptomic information encompassing non-coding RNAs regarding aging and rejuvenated mice [10]. Here, the authors were interested in identifying whether non-coding RNA expression changed across different organs and tissues and with age. Non-coding RNA sequencing was undertaken on 771 tissue samples of 16 different organ tissues at ten time points from 1 to 27 months of age. The total sequencing reads were ultimately mapped to 58,422 non-coding RNAs altogether, with 36.2% of sequenced reads across all tissues mapped to just miRNAs (microRNAs).

Across all tissues, it was seen that more miRNAs were correlated positively rather than negatively with age. It was also observed that miRNA pathways related to protein digestion and adsorption, metabolic pathways, and insulin resistance were enriched.

It was noted that specifically, miR-29c-3p displayed the most significant correlation with aging, with a generally increasing trend present across all tissues, plasma, and extracellular vesicles. It was also seen that for miR-29c-3p, there was a substantial rejuvenation following heterochronic parabiosis in mouse liver, with levels highlighting an organ-specific response to rejuvenation. MiR-29c-3p was also shown to regulate expression on gene targets involved in extracellular matrix-related processes. This supports the authors’ hypothesis that the miR-29 family is essential within organismal aging due to its regulatory nature on these target genes.

MicroRNAs, in addition to their roles in regulating gene expression, are also considered promising age-specific disease biomarkers, enhancing knowledge surrounding non-coding RNAs within major organs. This will aid in future progression towards successful targeted RNA-based therapies.

## 9. Shoot the Messenger! Small Interfering RNA (siRNA) Coming of Age

### Highlight by Alessandro Mannucci and Ajay Goel

Once dismissed as genomic “dark matter”, non-coding RNAs have emerged as pivotal players in cellular processes. Small interfering RNAs (siRNAs) disrupt gene expression by inducing messenger RNA degradation through RNA interference. The DICER enzyme processes double-stranded RNA into siRNA, which then binds to the RNA-induced silencing complex (RISC) and guides it to the target mRNA, ultimately cleaving the mRNA to impede translation.

In the REEF-1 trial, a novel siRNA treatment targeting transcripts from cccDNA and host-integrated HBV DNA (JNJ-3989) demonstrated the efficacy and safety for chronic hepatitis B, which affects 3.5% of the world population [11,12,13]. Current treatment options are either poorly tolerated or necessitate lifelong treatment with only a reduction—not elimination—of hepatocellular carcinoma risk and very low rates of functional cure (i.e., HBsAg seroclearance off-treatment). This multicenter, double-blind study randomized patients to six interventional arms for 48 weeks: conventional treatment ± siRNA at three dosages (40, 100, or 200 mg S.C. q4w) ± a capsid assembly modulator.

Up to 19% of the siRNA-treated patients met the pre-specified primary endpoint for discontinuation of nucleos(t)ide analogues (vs. 2% and 0% in other arms). Post-discontinuation, only 2/45 required nucleos(t)ide re-treatment, and the majority remained off-treatment with suppressed HBV-DNA levels, indicating a sustained response. The trial also demonstrated a siRNA dose-dependent reduction in HBsAg concentration: 98% of those receiving 200 mg achieved a 10-fold reduction in HBsAg, and 74% achieved a 100-fold reduction (mean reduction = 2.6 log_10_ IU/mL). Moreover, all siRNA-treated patients maintained a 10-fold reduction in HBsAg even 24 weeks after treatment discontinuation, signifying a legacy effect extending beyond the treatment period. While a functional cure was not reached, eight patients in the siRNA arm experienced transient seroclearance. This highlights the promise of siRNA in transforming treatment approaches, not only in hepatitis B but potentially also in other diseases, including cancer.
